# 全电视辅助胸腔镜手术治疗早期非小细胞肺癌

**DOI:** 10.3779/j.issn.1009-3419.2010.03.17

**Published:** 2010-03-20

**Authors:** Christopher Q. CAO, Stine MUNKHOLM-LARSEN, Tristan D. YAN, 炜 王, 文龙 邵

**Affiliations:** 1 广州医学院第一附属医院胸外科; 2 广州呼吸疾病研究所; 3 From Department of Cardiothoracic Surgery, Royal Prince Alfred Hospital and the Baird Institute for Applied Heart and Lung Surgical, University of Sydney, Sydney, NSW, Australia

**Keywords:** 电视辅助胸腔镜手术, VATS, 非小细胞肺癌, 肺叶切除术

## Abstract

从出现至今，微创手术对所有的外科分支学科均产生了巨大影响。电视辅助胸腔镜（VATS）肺叶切除术治疗早期非小细胞肺癌于20世纪90年代早期被首次报道，自此在许多医疗中心中逐渐普及。这项较新的技术的支持者列举了该技术在围手术期可能获得的许多有利结果，可能主要是因为该技术可减少手术创伤和应激反应。然而，由于仍缺乏有关长期生存和复发率的详实的临床数据，大部分胸心外科医生仍然持怀疑态度。

“全” VATS的定义目前仍不明确，之前的许多研究将其视为“小切口开胸肺叶切除术”，而非VATS肺叶切除术。在此，我们复习了有关全VATS肺叶切除术的文献，特别关注了直接对比VATS肺叶切除术和传统开胸肺叶切除术的比较研究。

自20世纪80年代晚期期第一例腹腔镜胆囊切除术以来，微创手术已经使外科的许多分支发生了变革。自20世纪90年代早期第一例电视辅助胸腔镜手术(VATS)肺叶切除术治疗早期非小细胞肺癌被多家研究机构同时报道^[1-3]^后，几项研究结果显示该新技术相关的潜在优势可能是因为该技术科减少术中创伤^[4-13]^(表-1)。时至今日，有两项随机对照试验(RCTs)已完成，且证实与传统开胸肺叶切除术相比，VATS肺叶切除术是安全、可行的^[1, 4]^。或许更为重要的是，最近的一项系统评价和*meta*分析显示，VATS肺叶切除术与传统开胸肺叶切除术在局部复发率方面无明显差别，而且VATS肺叶切除术可能与全身复发率的降低及5年总死亡率的改善有关^[14]^。通过这些报道，在过去十年中，特别是在一些大的医疗中心，VATS肺叶切除术应用的稳步增加已不让人感到吃惊^[15]^。

尽管VATS肺叶切除术取得了令人鼓舞的成绩，但必须意识到医疗机构间操作的不同质性，包括患者的筛选、肋骨牵开器或拉钩的使用以及切口长度等方面的显著差别。实际上，VATS的准确定义尚不明确，一些定义将其视为“电视辅助的小切口开胸术”，而不是完全不需要肋骨牵开器的VATS肺叶切除术^[4]^。作为反应，国际微创胸心外科协会在规范VATS定义方面做出了一致努力^[16]^。基于这些定义，仅少量非随机对照试验直接评估了全VATS技术治疗早期肺癌的疗效。在此，我们对有关VATS肺叶切除术的安全性和有效性的现有文献做一综述，特别关注非随机对照研究的结果。

## 安全性

### 围手术期死亡率和并发症发生率

同其它新外科技术一样，由于缺乏经验，一些可以避免的不良事件也可能会发生^[17]^。然而，根据可得数据，VATS肺叶切除术的围手术期死亡率极低。实际上，所有的对照研究均未发现VATS肺叶切除术与传统开胸肺叶切除术在术后死亡率方面存在显著差异(表 2)。此项发现在McKenna等^[18]^进行的包括1 100例接受VATS肺叶切除术患者的大型系列研究中得到进一步证实，该研究的术后死亡率可接受，仅为0.8%。

**1:** 有关直接比较早期非小细胞肺癌患者全电视辅助胸腔镜手术（VATS）与开放肺叶切除术的试验的总结

研究	年份	研究类型	*n*	分期	开口大小
Sugi *et al*.^[4]^	2000	RCT	100	cIA	8 cm
Sugiura *et al*.^[5]^	1999	OC	44	CIA (36), cIB (8)	6 cm
Inada *et al*.^[6]^	2000	OC	54	cIA+B	7 cm
Yim *et al*.^[7]^	2000	OC	36	cIA+B	NR
Koizumi *et al*.^[8]^	2002	OC	87	cIA+B	10 cm
Muraoka *et al*.^[9]^	2006	OC	85	cIA	8 cm
Sakuraba *et al*.^[10]^	2007	OC	140	cIA	5 cm
Petersen *et al*.^[11]^	2007	OC	100	pⅠ (40), pⅡ (24)	5 cm
Whitson *et al*.^[12]^	2007	OC	147	cIA+B	6 cm
Park *et al*.^[13]^	2007	OC	244	cIA+B	4 cm
RCT：随机对照试验；OC：观测队列研究；NR：未报道。 注：本表得到版权所有者© 2009 Journal of Thoracic Disease复制许可。					

**2:** 早期非小细胞肺癌实施电视辅助胸腔镜手术（VATS）或开胸肺叶切除术后术中结果及术后并发症

研究	死亡率		转换率	持续漏气			肺炎			心律失常			手术时间（h）			失血（mL）			胸管留置时间（d）			住院时间（d）	
	VATS	开胸术		VATS	开胸术		VATS	开胸术		VATS	开胸术		VATS	开胸术		VATS	开胸术		VATS	开胸术		VATS	开胸术
Sugi *et al*.^[4]^	NR	NR	4%	NR	NR		NR	NR		NR	NR		NR	NR		NR	NR		NR	NR		NR	NR
Sugiura *et al*.^[5]^	0/22	0/22	12%	2/22	3/22		2/22	0/22		NR	NR		3.8	3.3		150	300		NR	NR		23	22
Inada *et al*.^[6]^	NR	NR	NR	0/24	0/30		NR	NR		NR	NR		4.7	3.7		201	244		6.5	5.7		15.4	12.2
Yim *et al*.^[7]^	0/18	0/18	0%	1/18	1/18		NR	NR		NR	NR		1.3	1.4		NR	NR		3.2	4.1		4.1	5.3
Koizumi *et al*.^[8]^	0/52	0/35	NR	4/52	2/35		0/52	4/35		11/52	4/35		4.7	4.5		253	443		NR	NR		NR	NR
Muraoka *et al*.^[9]^	0/43	0/42	9%	1/43	3/42		1/43	1/42		2/43	10/42		4.8	4.9		151	362		3	3.9		NR	NR
Sakuraba *et al*.^[10]^	0/84	0/56	8%	NR	NR		NR	NR		NR	NR		NR	NR		NR	NR		NR	NR		NR	NR
Petersen *et al*.^[11]^	0/57	0/43	5%	NR	NR		1/57	3/43		8/57	3/43		NR	NR		NR	NR		3.1	4.7		4.2	5.3
Whitson *et al*.^[12]^	/59	/88	16%	8/59	10/88		2/59	17/88		8/59	9/88		3.8	3.5		251	255		5	6.1		6.4	7.7
Park *et al*.^[13]^	0/122	3/122	NR	4/122	7/122		NR	NR		15/122	20/122		3.7	3		NR	NR		NR	NR		4.9	7.2
NR：未报道。 注：本表得到版权所有者© 2009 Journal of Thoracic Disease复制许可。																							

并发症的发生率对病人的生存质量可产生重大影响，而且会影响患者的住院时间、身体机能和社会功能^[19]^。Muraoka等^[9]^和Park等^[13]^均指出，VATS肺叶切除术组并发症的总发生率明显低于开胸肺叶切除术组。或许与此有关，Petersen等^[11]^和Park等^[9]^也报道，VATS肺叶切除术组患者的住院时间显著缩短。而且，McKenna等^[18]^报道VATS肺叶切除术患者的平均住院时间少于5天^[19]^。

### 心律失常

房颤(AF)和其它类型的心律失常被认为与非心胸外科手术患者术后并发症发生率增加和住院时间延长相关^[20]^。Muraoka等^[9]^报道VATS肺叶切除组患者术后心律失常的发生率明显降低(RR=0.20, 95%CI: 0.05-0.84)，但尚无法阐明这一结果的具体原因。在VATS肺叶切除术组，输血所导致的继发性心脏负荷过重发生率的降低以及进行选择性淋巴结清扫时对迷走神经心脏分支的保护可能是一种合理的解释。相反，Park等^[13]^实施的一项大型研究对VATS肺叶切除术组的112名患者与传统开胸肺叶切除术组进行了匹配，但并未发现两组在术后AF发生率方面存在显著差别。他们认为，患者术后发生房颤的主要原因是由于肺切除引起的自主去神经和应激介导的神经体液机制，而非切口相关效应。其它的一些研究也证实，两种手术方式在术后心律失常的发生率方面无显著差异^[8, 11, 12]^。然而，由于各研究机构对心律失常的判定、预防策略以及监测技术不同，因此对这些结果应持谨慎态度。

### 肺炎

Whitson等^[12]^发现VATS肺叶切除术组中发生术后肺炎的患者极少(RR=0.18, 95%CI: 0.04-0.73)，且提示这可能与术后炎症减轻、疼痛减轻以及渗出减少的综合作用有关。尽管其它一些研究并未发现VATS组与开胸肺叶切除术组在肺炎发生率方面存在显著差异^[8, 11, 13]^，Muraoka等^[9]^发现两种手术方式在术后的痰液淤积方面存在显著差异(*p*=0.026)，并认为其与VATS肺叶切除术患者术后疼痛较轻有关，而且可使其它的呼吸并发症减少，包括肺不张和ARDS。

### 疼痛

术后疼痛控制对患者的恢复至关重要，且在术后护理中是必需的^[21]^。许多研究表明，微创技术与术后疼痛的减轻有关^[22, 23]^。Yim等^[7]^和Muraoka等^[9]^均报道VATS肺叶切除术组患者的术后疼痛等级降低。尽管病例数相对较少，但Yim等^[7]^发现VATS肺叶切除组患者对注射类麻醉品的需求量明显减少。同样，Muraoka等^[9]^通过硬膜外止痛泵的持续时间、附加止痛剂的需求量以及视觉模拟疼痛等级以评估术后的疼痛情况，研究发现VATS肺叶切除术组患者术后疼痛明显减轻。此外，他们发现，在研究中接受VATS肺叶切除术的所有患者术后第一天即可在重症监护室的床边站立。相反，开胸肺叶切除术组患者并不能获得此效果，即使两个组通过硬脊膜外布比卡因持续泵入而接受相同的疼痛控制方案。Muraoka进一步指出，这一发现对病人日常生活的早期恢复尤为重要。

### 炎症标志物

为支持VATS肺叶切除术给患者造成的手术创伤和应激较轻的假想，一些研究已对VATS组与开胸术组术后血清炎症标记物进行了比较。Yim等^[7]^发现VATS肺叶切除术组患者术后48 h IL-6和IL-8的水平显著降低。但是，研究也发现这些患者的抗炎细胞因子——IL-10的水平显著降低。综合这些结果，Yim认为开胸术可能与促炎介质和抗炎介质的失衡加剧有关，这主要是由于炎性损伤范围的增加所致。Muraoka等^[9]^记录了术后白细胞计数和C反应蛋白的最高水平，且发现接受VATS肺叶切除术的患者此两项指标水平明显降低。然而，应该指出的是，据Yim等的报道，他们并未发现术后第1天IL-6和IL-8的水平显著降低。

### 术中转为开胸术的情况

不同机构间VATS肺叶切除术向开胸肺叶切除术的转化率差别很大，从0%到16%不等^[4-13]^。争议的焦点为：接受VATS肺叶切除术失败后必须转为开胸术患者的分组问题。在一项RCT中，因无法安全分离叶间肺动脉或肺叶间不完全分离，Kirby等^[1]^将3名患者剔除出VATS肺叶切除术组。另一项由Sugi等实施的RCT最初将50例患者随机分组，但在统计分析时将术中出血需要转为开胸术的2例患者由VATS肺叶切除术组转至开胸术组。有人认为这样的病例转移并不公正，因VATS并发症而需转为开胸术的2例患者，仍应包括在VATS组^[4]^。

VATS肺叶切除术的支持者强调与微创技术相关的一些潜在的优势，包括术中出血的减少^[5, 9]^、胸管留置时间^[9]^和住院时间的缩短^[11]^。除生存质量的潜在改善外，这些因素在临床控制中也具有重要的价值。例如，Petersen等^[11]^发现，VATS肺叶切除术组患者对辅助化疗方案的依从性更好，且大部分患者可耐受更大剂量的化疗药物。这可能与术后并发症减少和患者可从VATS手术中更快恢复有关。

## 有效性

尽管越来越多的证据表明，VATS肺叶切除术与一些短期效应有关，如并发症发生率的减少、术后疼痛的减轻及康复加快，最终的问题为：在不损害生存期、局部和全身复发率等长期肿瘤学疗效的情况下，这些指标是否可实现。

一些比较研究发现，局部或全身复发率无显著差异^[4, 8, 10]^。研究结果见表 3。最近的一项*meta*分析^[14]^报道，与开胸术相比，VATS肺叶切除术的全身复发率降低(*p*=0.03)。

**3:** 早期非小细胞肺癌实施电视辅助胸腔镜手术（VATS）*vs*开胸肺叶切除术后患者长期生存、局部及全身复发率的总结

研究	所有原因的5年死亡率			局部复发率			全身复发率	
	有效率	95% CI		VATS	开胸术		VATS	开胸术
Sugi *et al*.^[4]^	0.90	0.29-2.77		3/48	3/52		2/48	7/52
Koizumi *et al*.^[8]^	0.75	0.30-1.89		2/45	4/32		4/45	7/32
Sakuraba *et al*.^[10]^	0.72	0.37-1.41		1/84	1/56		6/84	5/56
本表得到版权所有者© 2009 Journal of Thoracic Disease复制许可。								

与局部及全身复发率的研究结果相似，一些研究发现VATS肺叶切除术组与开胸肺叶切除术组间的5年死亡率无显著差别^[4, 8, 10]^。事实上，一项*meta*分析^[14]^显示，接受VATS肺叶切除术患者的5年死亡率较低(*p*=0.04)。

## 讨论

VATS肺叶切除术首次报道后不久，Kirby等^[1]^即进行了第一次RCT，以评估该项新技术的安全性与潜在的优势。这项试验共纳入已接受过VATS肺叶切除术或微创肌肉非损伤性开胸术的临床Ⅰ期NSCLC患者61例，研究发现VATS肺叶切除术组的术后并发症发生率显著降低(6% *vs* 16%)，但是在胸管留置时间、术后出血、住院时间或术后疼痛方面无显著降低。应当指出的是，在VATS组的所有患者均不可避免地使用了肋骨牵开器，且对数量不明确的患者实施了小切口开胸。Sugi等^[4]^进行的第二项RCT将100例临床IA期的肺癌患者随机分至VATS肺叶切除术与纵隔淋巴结清扫术组或后外侧切口开胸肺叶切除术组。该研究发现，VATS组与开胸术组在复发率和生存率方面无明显差异，其总的5年生存率分别为90%和85%。尽管这些研究报告的结果令人鼓舞，由于多种原因，此两项RCT被审核。第一，一些医生对VATS肺叶切除术的精确定义仍存质疑^[4]^。美国癌症和白血病研究组B (CALGB) 39802前瞻性试验^[24]^实施的一项多中心研究强调了“全” VATS肺叶切除术与小切口开胸术间的模糊界定，研究将VATS肺叶切除术定义为：在未使用拉钩或肋骨撑开器的情况下，逐个结扎叶间血管和支气管，并进行肺门淋巴结清扫术或取样。对该两项RCT的另一种批评意见是在统计分析时病人所在研究组的指定问题，因为这两项研究均将VATS肺叶切除术组中转而实施开胸术的患者进行了剔除或是转换。

除上述RCTs外，一些非随机对照研究已经开展，用以评估VATS与开胸肺叶切除术间的一些指标^[4-13]^。研究表明VATS方法具有一些潜在优势，包括心律失常、肺炎、术中出血、术后疼痛、炎症反应、胸管留置时间、住院天数、总的并发症均减少。总体而言，现有文献表明，在有资质的医疗中心，对于晚期NSCLC，VATS可完全代替开胸术治疗早期NSCLC，这与VATS可减少并发症有关，且对总生存期或复发率无影响。然而，目前仍缺乏全VATS肺叶切除术与传统开胸肺叶切除术进行直接比较的可靠的临床数据，且在有关恰当地界定患者对象的深入研究出现之前，很难阐明该项相对较新技术的优势。今后的研究应着重于募集样本量更大的患者，且最好是设计合理的RCT形式的研究。
